# A Multi-Modal Deep Learning Approach for Predicting Eligibility for Adaptive Radiation Therapy in Nasopharyngeal Carcinoma Patients

**DOI:** 10.3390/cancers17142350

**Published:** 2025-07-15

**Authors:** Zhichun Li, Zihan Li, Sai Kit Lam, Xiang Wang, Peilin Wang, Liming Song, Francis Kar-Ho Lee, Celia Wai-Yi Yip, Jing Cai, Tian Li

**Affiliations:** 1 Department of Health Technology and Informatics, The Hong Kong Polytechnic University, Hong Kong SAR, China; zhichun20.li@connect.polyu.hk (Z.L.); zh-amber.li@connect.polyu.hk (Z.L.); albert-xiang.wang@connect.polyu.hk (X.W.); peilin.wang@connect.polyu.hk (P.W.); li-ming.song@connect.polyu.hk (L.S.); 2 Department of Biomedical Engineering, The Hong Kong Polytechnic University, Hong Kong SAR, China; saikit.lam@polyu.edu.hk; 3 Research Institute for Smart Ageing, The Hong Kong Polytechnic University, Hong Kong SAR, China; 4 Department of Clinical Oncology, Queen Elizabeth Hospital, Hong Kong SAR, China; leekh4@ha.org.hk (F.K.-H.L.); ywy923@ha.org.hk (C.W.-Y.Y.)

**Keywords:** nasopharyngeal carcinoma, adaptive radiation therapy, convolutional neural network, vision transformers, medical image classification

## Abstract

Background: Adaptive radiation therapy (ART) can improve prognosis for nasopharyngeal carcinoma (NPC) patients. However, the inter-individual variability in anatomical changes, along with the resulting extension of treatment duration and increased workload for the radiologists, makes the selection of eligible patients a persistent challenge in clinical practice. The purpose of this study was to predict eligible ART candidates prior to radiation therapy (RT) for NPC patients using a classification neural network. By leveraging the fusion of medical imaging and clinical data, this method aimed to save time and resources in clinical workflows and improve treatment efficiency. Methods: We collected retrospective data from 305 NPC patients who received RT at Hong Kong Queen Elizabeth Hospital. Each patient sample included pre-treatment computed tomographic (CT) images, T1-weighted magnetic resonance imaging (MRI) data, and T2-weighted MRI images, along with clinical data. We developed and trained a novel multi-modal classification neural network that combines ResNet-50, cross-attention, multi-scale features, and clinical data for multi-modal fusion. The patients were categorized into two labels based on their re-plan status: patients who received ART during RT treatment, as determined by the radiation oncologist, and those who did not. Results: The experimental results demonstrated that the proposed multi-modal deep prediction model outperformed other commonly used deep learning networks, achieving an area under the curve (AUC) of 0.9070. These results indicated the ability of the model to accurately classify and predict ART eligibility for NPC patients. Conclusions: The proposed method showed good performance in predicting ART eligibility among NPC patients, highlighting its potential to enhance clinical decision-making, optimize treatment efficiency, and support more personalized cancer care.

## Simple Summary

Nasopharyngeal carcinoma (NPC) is a type of cancer that often requires radiation therapy as a primary treatment. During therapy, some patients may experience anatomical changes that make adaptive radiation therapy (ART) necessary to improve treatment outcomes. However, identifying which patients will need ART is usually carried out only after treatment has started, which can be time consuming and resource intensive. In this study, we developed a deep learning model that combines medical imaging and clinical data to predict ART eligibility before treatment begins. This early prediction has the potential to help clinicians to better plan therapy in advance, reduce unnecessary delays, and make more efficient use of medical resources. By identifying suitable ART candidates ahead of time, our approach has the potential to improve personalized cancer care and support faster clinical decision-making.

## 1. Introduction

Nasopharyngeal carcinoma (NPC) is a highly aggressive tumor of the head and neck, and the most prevalent cancer originating in the nasopharynx which is an anatomical region located at the posterior part of the nasal cavity connected to the pharynx. The incidence of NPC varies significantly across the globe, with the highest prevalence observed in populations from southern China and Southeast Asia [1]. Due to the close proximity of NPC tumors to vital organs and cervical lymph nodes, surgical intervention is seldom used. Instead, radiotherapy (RT), particularly intensity modulated radiation therapy (IMRT), has become the standard treatment modality for NPC management [2,3,4]. IMRT enables precise targeting of tumors while minimizing exposure to surrounding healthy tissues. However, anatomical changes that occur during radiation therapy, such as weight loss and tumor shrinkage [5,6], may affect dose distribution and compromise treatment accuracy [7,8]. Adaptive radiotherapy (ART) offers a solution by using image-guided updates to account for anatomical variations and modify the treatment plan, improving tumor control and reducing toxicity to critical structures [9]. Anatomical changes, along with dosimetric deviations, imaging findings, and clinical responses are important factors considered in the evaluation of whether adaptive radiotherapy is necessary, and the influence of these factors also makes it difficult to establish a standardized ART protocol that can be universally adopted. Simultaneously, implementing ART is resource-intensive, requiring substantial manpower, imaging time, and manual intervention [10,11]. Therefore, developing automated classification methods to identify ART eligible patients prior to treatment is critical for improving clinical efficiency and optimizing resources.

Several studies have investigated ART eligibility prediction using features extracted from clinical images [12,13,14]. These traditional methods rely on handcrafted radiomic features extracted from the computed tomographic (CT) and magnetic resonance imaging (MRI) scans, such as first-order, shape, and texture features. For example, Yu et al. [12] applied least absolute shrinkage and selection operator-based logistic regression to radiomic features from pre-treatment MRI images and achieved area under the curve (AUC) values ranging from 0.75 to 0.93. Lam et al. [14] investigated the predictive role of radiomic features extracted from pre-treatment CT, MRI, organ contours, and dose distributions, applying ridge regression and multi-kernel learning for classification, and subsequently developed multiple multi-omics models. However, these handcrafted feature-based methods have some limitations. They heavily rely on manually engineered features, which may fail to capture the complex spatial structures and high-level semantic information present in medical images; thus, effectively utilizing improved feature representation to accurately predict tumor variability remains a persistent challenge [15]. Additionally, radiomic features are sensitive to variations in imaging acquisition parameters, reconstruction algorithms, and preprocessing procedures, which compromises reproducibility. Moreover, traditional methods often involve multi-stage processing pipelines, making end-to-end optimization challenging.

Deep learning techniques like convolutional neural networks (CNNs) have been widely applied for classification tasks, providing potential solutions for medical diagnosis and analysis [16]. These networks can automatically extract complex and hierarchical features from medical imaging data, which enables them to achieve better performance than the traditional methods [17]. The Vision Transformer (ViT) [18] and CNNs have emerged as two leading architectures for image analysis. Specifically, architectures such as Inception v1–v4 [19,20], ResNet [21], and DenseNet [22] have demonstrated strong performance in medical image diagnostics. Specifically, Inception networks progressively integrate residual connections and batch normalization techniques, while ResNet addresses the vanishing gradient problem through skip connections. DenseNet enhances learning by interconnecting all layers to promote feature reuse. ViT relies on self-attention mechanisms and has shown competitive performance in medical imaging, although it typically requires large datasets for effective training, which is a significant challenge in the medical domain [23]. Additionally, feature fusion and multi-modal learning have garnered increasing research interest as effective means to improve predictive accuracy. Feature fusion strategies can compensate for missing information and enhance the expressiveness of deep models [24]. Among these approaches, feature-level fusion has demonstrated consistently strong performance, outperforming both classifier-level and decision-level fusion [25]. Multi-modal models can form more comprehensive representations [26]. Particularly, multi-scale fusion techniques, such as feature pyramid networks [27], improve feature representation by hierarchically integrating semantic information across multiple layers that possess varying spatial resolutions. Additionally, attention mechanisms offer the capability to dynamically modulate feature responses based on contextual relevance, which allows neural networks to prioritize the most informative components of the input data [28]. Several attention modules have been proposed to operationalize this mechanism, including the convolutional block attention module [29], the squeeze-and-excitation module [30], and dual attention networks [31], all of which have been incorporated into deep learning frameworks to enhance the internal representation of features through both channel-level and spatial-level reweighting strategies. When compared to conventional machine learning methods, deep models that incorporate attention mechanisms tend to exhibit significantly improved performance [32]. Furthermore, the cross-attention-based transformer employs a cross-attention strategy [33] that enables the model to simultaneously capture and integrate diverse feature representations derived from multiple modalities in the task of multi-modal learning, which contributes to more comprehensive feature interaction and more effective fusion across modalities.

Effectively integrating information from multiple modalities is a fundamental challenge in multi-modal learning. Traditional fusion strategies, such as early fusion and late fusion, have been widely adopted in previous work. Early fusion approaches concatenate low-level features from different modalities at the input stage, enabling joint modeling but often suffer from information redundancy and are sensitive to missing or noisy modalities. Late fusion merges predictions from independently trained modality-specific models, which increases robustness to modality-specific noise, but lacks the ability to capture cross-modal interactions during representation learning [34]. In contrast, the cross-attention strategy allows models to learn complex semantic alignments between modalities. This flexible and adaptive fusion enables more expressive multi-modal representations, which has the potential to address the limitations of static early or late fusion strategies [35]. Therefore, the cross-attention strategy could provide an effective proposal for multi-modal integration, making it a flexible choice for scenarios where capturing intricate inter-modal relationships is essential.

The aim of this study is to enhance the accuracy of ART eligibility prediction for patients with NPC from multi-modal data by developing a deep learning model. Specifically, the proposed model utilizes ResNet as the backbone network and incorporates multiple modules to process features across different imaging modalities, multi-scale image representations, and clinical data, thereby capturing complementary information. This end-to-end method is designed to enhance predictive accuracy and provide radiation oncologists with an effective auxiliary diagnostic tool, reducing the demand on medical resources and promoting more individualized ART workflows.

## 2. Materials and Methods

### 2.1. Dataset

The patient data utilized in this study consists of 305 NPC patients who received radiotherapy (RT) at Queen Elizabeth Hospital in Hong Kong for retrospective analysis. Patients included in this study were those with biopsy-confirmed primary NPC without distant metastasis or other concurrent malignancies at diagnosis, who received helical tomotherapy. Exclusion criteria included lack of administration of contrast agents for planning contrast-enhanced CT or MRI, or incomplete clinical and imaging data. Therefore, each patient had imaging data including CT images, pre-treatment contrast-enhanced T1-weighted (CET1-w), T2-weighted (T2-w) MR images, and clinical data. We collected clinical data including demographic information (gender, age, BMI), tumor characteristics (T stage, N stage, histological subtype), and tumor volume, as shown in Table 1. Patients were categorized into two groups based on their replan status—whether or not they received ART during their course of RT, as determined by the radiation oncologist. Patients who underwent ART were labeled as positive samples, while those who did not were labeled as negative samples.

### 2.2. Data Preprocessing

As an initial step in data preparation, CT and MRI images were registered to ensure spatial alignment. As CNNs operate solely in voxel space for image analysis tasks, they may overlook the actual physical size information present in the real-world spatial domain. All CT and MRI images were resampled to a voxel spacing of 1 × 1 × 1 mm^3^ to address this issue. For each case, CT and MRI images were cropped into volumetric region of interest patches with dimensions of 196 × 128 × 128, encompassing the primary tumor as well as the ipsilateral and contralateral parotid glands. These regions were then normalized using Z-score normalization and served as input for subsequent analysis. Additionally, the continuous variables in the clinical data (age, BMI, and tumor volume) were standardized. The dataset was split into training and testing sets in an approximate 8:2 ratio. Specifically, there were 180 negative cases and 53 positive cases in the training set, and 52 negative cases and 20 positive cases in the testing set. To increase the robustness of the model and the reliability of the results, we randomly select the train set three times and report the average result from three test sets. For avoiding overfitting, data augmentation techniques [36], including flipping, noise addition, and rotation, were applied to the training dataset.

### 2.3. Model Architecture

**Figure 1:**
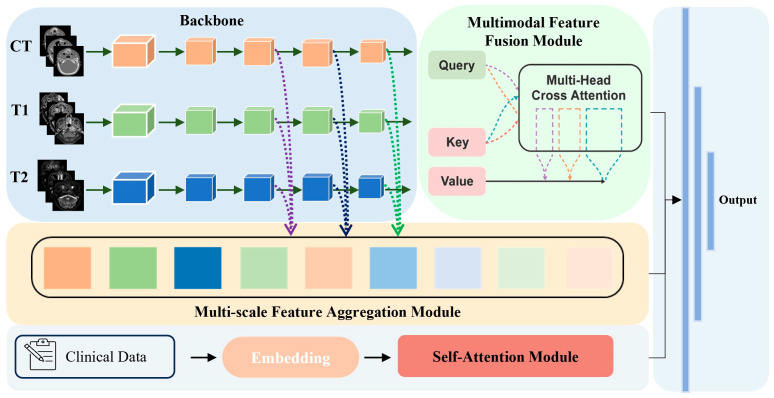
Overview of the proposed method.

In this study, we designed a multi-modal deep feature learning network to predict patient eligibility by incorporating medical images and clinical data, as illustrated in Figure 1. The features extracted from the shared parameters backbone were passed into the multi-modal feature fusion module, which combined information from different modalities by reallocating weights and highlighting key features. To further enhance feature representation, the multi-scale feature aggregation module was utilized to integrate features from different stages of the backbone network. Additionally, the self-attention module was applied to capture correlations among clinical variables, thereby assisting in outcome prediction.

#### 2.3.1. Backbone and Multi-Modal Feature Fusion Module

**Figure 2:**
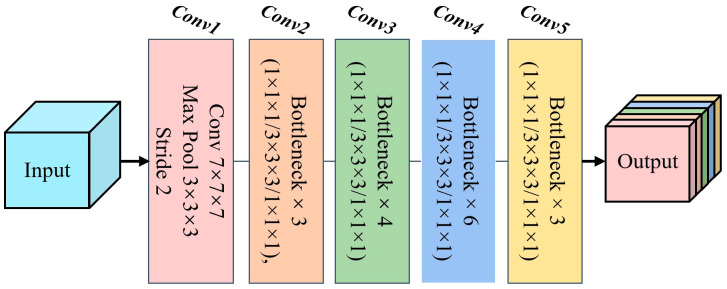
Schematic of the architecture of the backbone network.

The backbone of our network adopted the ResNet-50 architecture as the image feature extractor, which consisted of five main blocks (conv1 to conv5, as shown in Figure 2). We leveraged pre-trained weights from the MedicalNet [37] to prevent overfitting during training. This strategy allowed the model to utilize knowledge learned from a large-scale dataset, thereby improving feature representation [38]. Specifically, conv1 served as a preprocessing step for the input image, while conv2 through conv5 were composed of bottleneck structures with a similar design. Each bottleneck block consisted of three convolutional layers that performed dimensionality reduction, captured spatial information, and restored feature map dimensions to provide higher-level abstract representations. Batch normalization and non-linear activation functions were applied after these convolutional layers to enhance the representational capacity of the network and reduce sensitivity to variations in the input data distribution.

**Figure 3:**
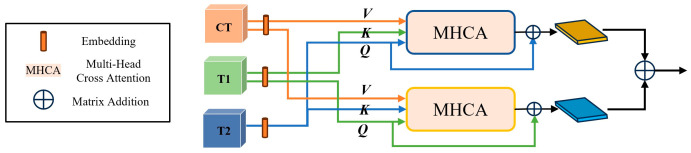
Multi-modal feature fusion module for images features.

Subsequently, the structure of our multi-modal feature fusion module was illustrated in Figure 3. After extracting high-level features from each image modality using the backbone network, we first embedded the features and then utilized two cross-attention-based transformers to perform deep cross-modal interaction. While cross-attention-based transformers typically assigned the query (Q) from one modality and the key and value (K, V) from another, we assigned Q, K, and V to three different modalities in each branch, as inspired by recent work [39,40,41], to fully exploit the complementary and high-order relationships among different image types. Specifically, we selected MRI modalities (CET1-w and T2-w image features), which may contain predictive biomarkers for tumor shrinkage following cancer treatment, as queries in the two branches, while using CT features as the value. This approach allowed the model to actively focus on the relevant information from MRI while still integrating complementary anatomical details from CT images. Specifically, the cross-attention output, denoted as $M_{Q\_T1}$, was mathematically expressed as:

(1) $${M_{Q\_T1}=SoftMax\left(\frac{{QK}^{T}}{\sqrt{d}}\right)V+F}_{T1}$$

where Q = $F_{T1}W_{Q}$, K = $F_{T2}W_{K}$, V = $F_{CT}W_{V}$, *d* is the dimension of Q, K,V features, and $W_{Q}$, $W_{K}$, $W_{V}$ are learnable weights. The final output of the cross-attention operation is computed as the sum of the attention result and the original input features. For the other cross-attention branch output, $M_{Q\_T2}$, we exchange $F_{T1}$ and $F_{T2}$ as Q and K. Then, the output feature maps from these two branches were subsequently aggregated via element-wise matrix addition.

#### 2.3.2. Multi-Scale Feature Aggregation and Self-Attention Modules

**Figure 4:**
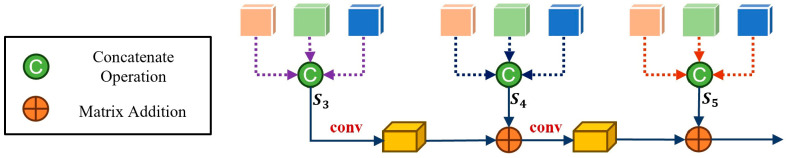
Structure of multi-scale feature aggregation module.

Building on the features extracted at different stages of the backbone, we introduced the multi-scale feature aggregation module shown in Figure 4 to further enhance modality integration. At each selected stage of the backbone network (conv3, conv4, and conv5), we obtained feature maps corresponding to the three imaging modalities. For each stage, these feature maps were concatenated, which preserves the unique information from each modality and prevents early loss of modality-specific features. A convolutional layer with a kernel size of 1 was then applied to each concatenated map to reduce the channel dimension. To achieve effective multi-scale fusion, the fused feature maps from different stages were further aggregated. Specifically, the output of each convolutional operation was combined with the corresponding feature map from the next stage through element-wise addition, which can be formulated as:

(2) $$I_{msf}=Add\left(conv\left(Add\left(conv\left(S_{1}\right),S_{2}\right)\right),S_{3}\right)$$

where $I_{msf}$ denotes the final output of the multi-scale feature aggregation module, $S_{m}=Cat\left(f_{i}^{j}\right)$, with $f_{i}^{j}$ representing the feature map of modality  i at stage j ( $i\;\in \;\left\{CT,\;T1,T2\right\}$, $j\in \;\left\{3,\;4,\;5\right\}$); Cat denotes the concatenation operation, and conv denotes the convolution operation. This hierarchical aggregation strategy enabled the module to integrate the information from different receptive fields, thereby enhancing the representational capacity of the network.

Alongside the image features, the clinical data were first preprocessed such that categorical variables (gender, T stage, N stage, histological subtype) were transformed into binary vectors using one-hot encoding, thereby converting each category into a distinct binary feature. Continuous variables (age, BMI, and tumor volume) were included in their standardized form, as previously described. The resulting clinical feature vector was then input into a transformer-based module composed of three stacked encoder layers, each containing a single-head self-attention mechanism and a feed-forward neural network. The final output vector of this module was subsequently passed through fully connected layers to generate the final prediction.

### 2.4. Implementation Details and Evaluation Metrics

All experiments were implemented using PyTorch 2.0.1 on a Windows 10 operating system. The training and evaluation procedures were conducted on a workstation equipped with an Intel(R) Core (TM) i9-13900 CPU (Intel Corporation, Santa Clara, CA, USA) and an INNO3D RTX 4090 GPU (InnoVISION Multimedia Limited, Hong Kong). Table 2 shows the summary of hyperparameters applied for this model. The Adam optimizer was used for parameter optimization, with the initial learning rate set to 1 × 10^−3^. A StepLR learning rate scheduler was applied, where the learning rate was decayed by a factor of 0.9 every five epochs. And the cross-entropy loss function was used. The batch size for training was set to 2, and all models were trained for a maximum of 150 epochs.

We evaluated model performance using four classification metrics: sensitivity, specificity, accuracy, and AUC. Sensitivity measures the proportion of actual positive cases correctly identified by the model, while specificity refers to the proportion of actual negative cases correctly identified. Accuracy quantifies the overall correctness of the model’s predictions. AUC reflects the overall ability of the model to distinguish between positive and negative cases. The formulas for these evaluation metrics are as follows:

(3) $$Sensitivity=\frac{TP}{TP+FN}$$

(4) $$Specificity=\frac{TN}{TN+FP}$$

(5) $$Accuracy=\frac{TP+TN}{TP+FN+TN+FP}$$

where *TP* denotes correctly classified positive samples; *FN* denotes positive samples incorrectly predicted as negative; *FP* denotes negative samples incorrectly predicted as positive; *TN* denotes correctly classified negative samples.

## 3. Results

### 3.1. Comparison with Other Deep Learning Networks

We conducted a comparison between our proposed network and several deep learning models that use only images as input, including DenseNet121, ResNet50, SE-ResNet50, BoTNet18, and ViT. DenseNet121 represents a classic densely connected convolutional network, SE-ResNet50 integrates squeeze-and-excitation modules into ResNet50 to recalibrate channel-wise feature responses, BoTNet18 replaces the standard 3 × 3 convolution with multi-head self-attention to enhance global context modeling, and ViT adopts transformer-based architecture for image representation learning. For all these models, images from multiple modalities were provided as input, ensuring a fair comparison with our proposed method.

The comparative results across all evaluation metrics are summarized in Table 3. DenseNet121 and ResNet50 exhibited relatively lower sensitivities, with values of 0.6377 and 0.6806, respectively, suggesting a higher rate of missed positive cases. SE-ResNet50 and BoTNet18 showed moderate performance in sensitivity and specificity, achieving 0.6778 and 0.6611 for sensitivity and 0.8831 and 0.8767 for specificity, but neither surpassed ViT, which achieved 0.7095 for sensitivity and 0.8962 for specificity. However, our image branch attained the highest sensitivity and specificity, reaching 0.7579 and 0.9157, respectively, indicating improved detection of positive cases and more reliable exclusion of negatives. In terms of accuracy, our model also led with 0.8704, outperforming ViT at 0.8426 and all other baseline models, which ranged from 0.8009 to 0.8241. In addition, our approach achieved the highest AUC of 0.8908, reflecting the best overall discriminative capability among all models. These results demonstrate that our image branch achieves the most effective balance of sensitivity and specificity, as well as the highest overall accuracy and AUC, underscoring its clinical potential for medical image classification.

### 3.2. Ablation Study

Clarifying the contribution of each module is important for validating the overall network design; therefore, ablation experiments were performed to assess both individual and combined effects. For consistency and fairness, all experiments used ResNet50 as the backbone with identical pretrained weights for initialization. The results for each experimental configuration are shown in Table 4. In this table, Block1 denotes the multi-modal feature fusion module, Block2 means the multi-scale feature aggregation module, and Block3 presents the self-attention module.

We first analyze the results from the perspective of AUC to compare the performance improvements contributed by each module. Model1, which uses ResNet50 as the backbone network and takes the concatenation of images as input, achieves an AUC of 0.8215. The introduction of single modules in Model2, Model3, and Model4 produces observable enhancements. Specifically, Model2 utilizes the multi-modal feature fusion module to integrate features from different modalities and achieves a higher AUC value (0.8498), effectively supplementing and fusing high-level representations from various sources. Similarly, by employing the multi-scale feature aggregation module to integrate image features from multiple receptive fields, Model3 achieves better results than Model1. Although the addition of clinical data features provides a relatively smaller improvement (Model4), this module supplements imaging features with clinically relevant information. Furthermore, the combination of modules in Model5, Model6, and particularly Model7, which attains an AUC of 0.8908, further improves performance, highlighting the synergistic effects of integrating different modules. Specifically, the joint use of the multi-modal feature fusion module and the multi-scale feature aggregation module enables the model to pay attention to both local and global features of the images, enhancing its ability to capture relationships at different levels of representation. Finally, Model8 is the proposed method, and it achieves the highest AUC of 0.9070, validating the effectiveness of the proposed architecture.

**Figure 5:**
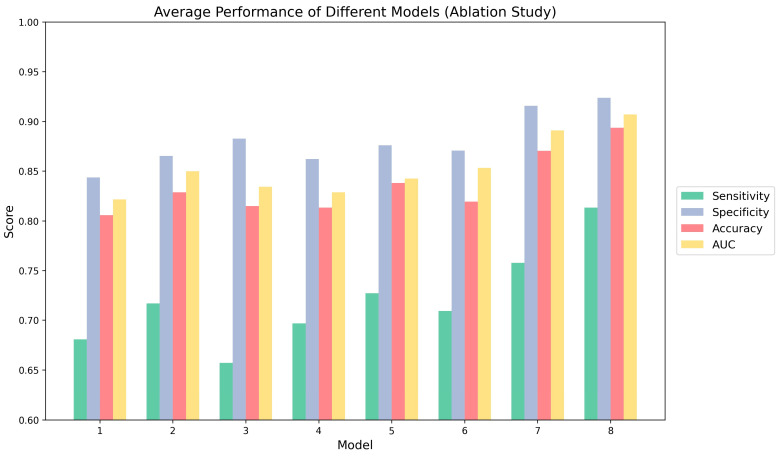
Performance of different models in ablation study.

Moreover, as illustrated in Figure 5, the following observations can be made: (1) The sensitivity results show a consistent upward trend with the addition of modules. It is worth noting that when the multi-scale feature aggregation module (Model3) is added alone, sensitivity decreases compared to the baseline (from 0.6806 in Model1 to 0.6570 in Model3). This may be due to the emphasis of this module on multi-scale feature extraction. While enhancing overall feature diversity, it may also lead to the dilution of highly discriminative features necessary for correctly identifying positive cases. However, this negative impact is mitigated when combined with other modules, and overall sensitivity is significantly improved. (2) Specificity and accuracy also improve, with specificity rising from 0.8435 to 0.9237 and accuracy from 0.8056 to 0.8935. These findings collectively highlight that module addition and combination can enhance model performance to an extent, improving the discriminative ability of the model. (3) Further comparison of Model7 and our proposed model, Model8, reveals that the inclusion of clinical data increases AUC by 1.82% and sensitivity by 7.29%. This demonstrates that clinical features can provide some valuable complementary information for classification. Although the contribution of clinical data may be limited by its lower dimensionality compared to image features, incorporating it with the self-attention mechanism improves model performance.

## 4. Discussion

The application of ART in NPC is important for maximizing treatment outcomes while minimizing toxicity to normal tissues. The prediction of ART eligibility can reduce clinical workload and hospital resource consumption by enabling personalized adjustments to the patient’s treatment strategy in a timely manner. In this study, we developed and evaluated a deep learning method. Deep learning enables direct prediction learning and can automatically extract features from images, independent of subjective feature engineering. Deep learning models can reduce the complexity of building multi-stage processing pipelines and do not rely on pre-defined features or rules, thus simplifying the learning process and allowing direct optimization for the final task.

The proposed method in this study showed good potential for predicting ART eligibility in NPC patients. The performance of the proposed model was evaluated and analyzed through comparative and ablation experiments. Our experiments showed that the proposed network can perform multi-modal fusion well and, compared with other deep learning models, our model showed a significant improvement in sensitivity and specificity, achieving an accuracy of 0.8935 and an AUC of 0.9070. The ablation studies showed that the multi-modal feature fusion module using cross-attention-based transformers contributed most significantly to the overall improvement of the model. Additionally, the use of multi-scale feature aggregation, which integrates image features from different receptive fields, and the incorporation of clinical data can also enhance the performance.

The decision to initiate adaptive radiotherapy in clinical practice is typically based on observable changes such as tumor shrinkage or alterations in patient contour during treatment. Our method can automatically extract representative features from both imaging and clinical data, allowing for more objective and accurate identification of patients who would benefit from ART. By capturing subtle yet clinically meaningful changes that may signal the need for treatment replanning, our model enhances its predictive ability and has the potential to optimize resource utilization in radiotherapy departments.

However, this study has several limitations. Firstly, our dataset was obtained from a single medical center, and there exists an imbalance between positive and negative cases in our sample. The patient sample distribution may differ across institutions, potentially impacting the generalizability. In addition, the ground truth labels for ART necessity in this study were determined retrospectively based on clinical decisions made by radiation oncologists, lacking a certain degree of standardization or objectivity in the criteria. This approach introduces potential subjectivity, raising the risk that the model may learn local clinical preferences rather than universally applicable ART indications. Addressing this limitation in future work through the adoption of standardized criteria and multi-institutional datasets will be essential to improve the objectivity and external generalizability of the model. Secondly, the selection of key hyperparameters was based on prior work and preliminary experiments rather than ablation studies. While these choices are consistent with established practices in the field, more comprehensive hyperparameter tuning in future studies may further improve model performance. Thirdly, another limitation of our study is the lack of model interpretability tools. The standard methods such as Grad-CAM and SHAP are not readily applicable or lack standardized solutions in this setting due to the complexity of our multi-modal deep learning architecture. However, as explainable AI methods for multi-modal models continue to develop, we plan to try to explore and incorporate these tools in future work to improve transparency and clinical trust. And future work may also consider incorporating additional modalities, such as patient dosimetry maps, which have been shown in previous studies to be important factors in radiotherapy planning and outcome prediction [42,43]. Integrating such data may help the model better capture patient characteristics and treatment responses. In addition, some research has demonstrated the effectiveness of leveraging both image and text information for improved medical prediction tasks [44,45]. While our current method regards clinical data and imaging features as separate branches, more advanced fusion strategies could be explored and used in the future.

## 5. Conclusions

Our proposed multi-modal classification network achieved promising results in predicting the eligibility of NPC patients for ART. By addressing the challenge of adaptive radiotherapy eligibility screening, our method can help clinicians more efficiently identify patients who are likely to benefit from ART, thereby enabling timely and individualized treatment adjustments. This has the potential to improve patient outcomes and optimize resource allocation within clinical practice. To explore the interconnectivity between multi-modal data, we employed attention-based methods and multi-scale feature fusion approaches to effectively combine local and global features from diverse modalities. Experimental results demonstrated that the proposed method achieved satisfactory classification performance for ART eligibility compared to other deep learning methods. Future research should focus on expanding the dataset and optimizing the model architecture, particularly to enhance computational efficiency and adaptability in resource-limited environments. Moving forward, actionable next steps include conducting prospective trials to validate the clinical utility of the model. Additionally, future research should focus on integrating standardized ART criteria, expanding the dataset to encompass more diverse patient populations, and further optimizing the model architecture. Future work should also explore novel multi-modal fusion strategies, particularly leveraging advances in foundation models, which hold significant potential for enhancing representational power and adaptability in clinical applications. In general, the proposed method provides a potential solution for developing an automatic auxiliary diagnosis system suitable for clinical adoption, which can help to implement individualized treatment strategies for patients.

## Figures and Tables

**Table 1:** Description of clinical data used in this study for ART eligibility prediction.

Parameter	Description	Data Acquisition
Gender	0 = Female	Demographic
	1 = Male	
Age	Patient age (Years)	Demographic
BMI	Patient body mass index	Demographic
T stage	1 = Tumor confined to nasopharynx or oropharynx/nasal cavity	Classified in the hospital
	2 = Parapharyngeal extension	
	3 = Invasion of bony structures or paranasal sinuses	
	4 = Intracranial extension and/or involvement of cranial nerves, hypopharynx, orbit, or infratemporal fossa	
N stage	1 = Unilateral lymph node(s), ≤6 cm, above the supraclavicular fossa	Classified in the hospital
	2 = Bilateral or contralateral lymph nodes, ≤6 cm	
	3 = Lymph node(s) >6 cm and/or involvement of supraclavicular fossa	
Histological subtype	1 = Keratinizing squamous cell carcinoma	Classified in the hospital
	2 = Differentiated keratinizing differentiated carcinoma	
	3 = undifferentiated carcinoma	
Tumor volume	Estimated value	Estimated from the longest and shortest diameter of the tumor.

**Table 2:** Hyperparameters of the proposed method used for the ART eligibility prediction.

Hyperparameter	Value
Batch size	2
Maximum epochs	150
Initial learning rate	1 × 10^−3^
Optimizer	Adam
Learning rate scheduler	StepLR
Step size	5
Learning rate decay rate	0.9
Loss function	Cross-entropy

**Table 3:** Performance comparison between the proposed method and other classic models.

Models	Sensitivity	Specificity	Accuracy	AUC
DenseNet121	0.6377	0.8545	0.8009	0.7521 (0.7381–0.7661)
ResNet50	0.6806	0.8435	0.8056	0.8215 (0.8172–0.8258)
SE-ResNet50	0.6778	0.8831	0.8241	0.8266 (0.8055–0.8477)
BoTNet18	0.6611	0.8767	0.8148	0.8304 (0.8219–0.8389)
ViT	0.7095	0.8962	0.8426	0.8359 (0.8191–0.8527)
Our Image Branch	0.7579	0.9157	0.8704	0.8908 (0.8873–0.8943)

**Table 4:** Ablation study results of the proposed method.

Model	Modules			Sensitivity	Specificity	Accuracy	AUC
	Block1	Block2	Block3				
1	**×**	**×**	**×**	0.6806	0.8435	0.8056	0.8215 (0.8172–0.8258)
2	✓	**×**	**×**	0.7168	0.8651	0.8287	0.8498 (0.8445–0.8548)
3	**×**	✓	**×**	0.6570	0.8826	0.8148	0.8342 (0.8313–0.8371)
4	**×**	**×**	✓	0.6967	0.8620	0.8132	0.8287 (0.8262–0.8312)
5	**×**	✓	✓	0.7271	0.8758	0.8380	0.8425 (0.8366–0.8484)
6	✓	**×**	✓	0.7093	0.8705	0.8194	0.8531 (0.8485–0.8577)
7	✓	✓	**×**	0.7579	0.9157	0.8704	0.8908 (0.8873–0.8943)
8	✓	✓	✓	0.8132	0.9237	0.8935	0.9070 (0.9047–0.9093)

## Data Availability

The data that support the findings of this study are available from the corresponding author upon reasonable request.
